# Supplementary figures and images for: Creating cell-specific computational models of stem cell-derived cardiomyocytes using optical experiments
Source: PLoS Comput Biol. 2024 Sep 11;20(9):e1011806. doi: 10.1371/journal.pcbi.1011806 (PMC11460686; doi:10.1371/journal.pcbi.1011806)

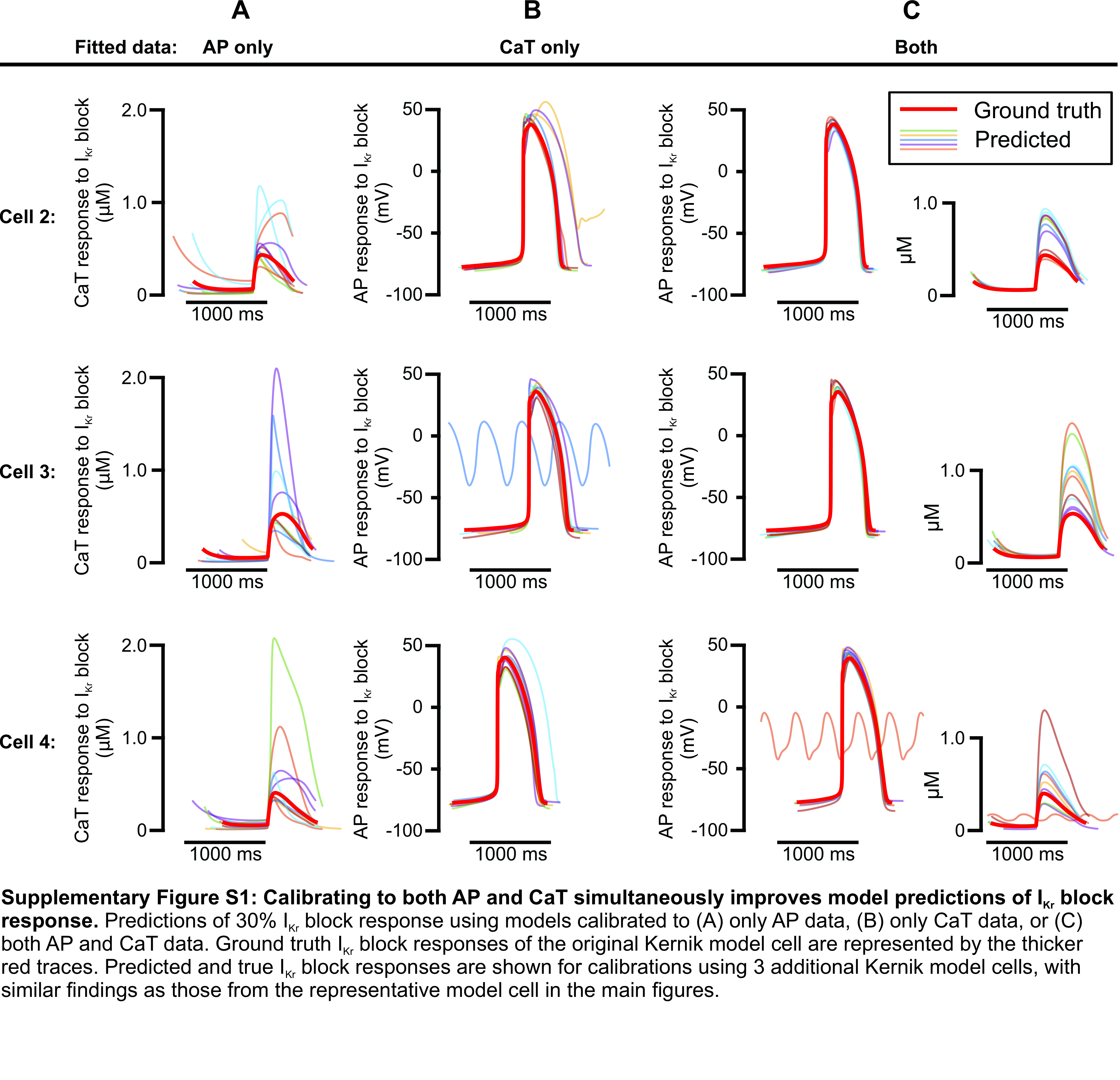

Supplement: S1 Fig — Predictions of 30% IKr block response using models calibrated to (A) only AP data, (B) only CaT data, or (C) both AP and CaT data. Ground truth IKr block responses of the original Kernik model cell are represented by the thicker red traces. Predicted and true IKr block responses are shown for calibrations using 3 additional Kernik model cells, with similar findings as those from the representative model cell in the main figures. (TIF) [file pcbi.1011806.s001.tif]

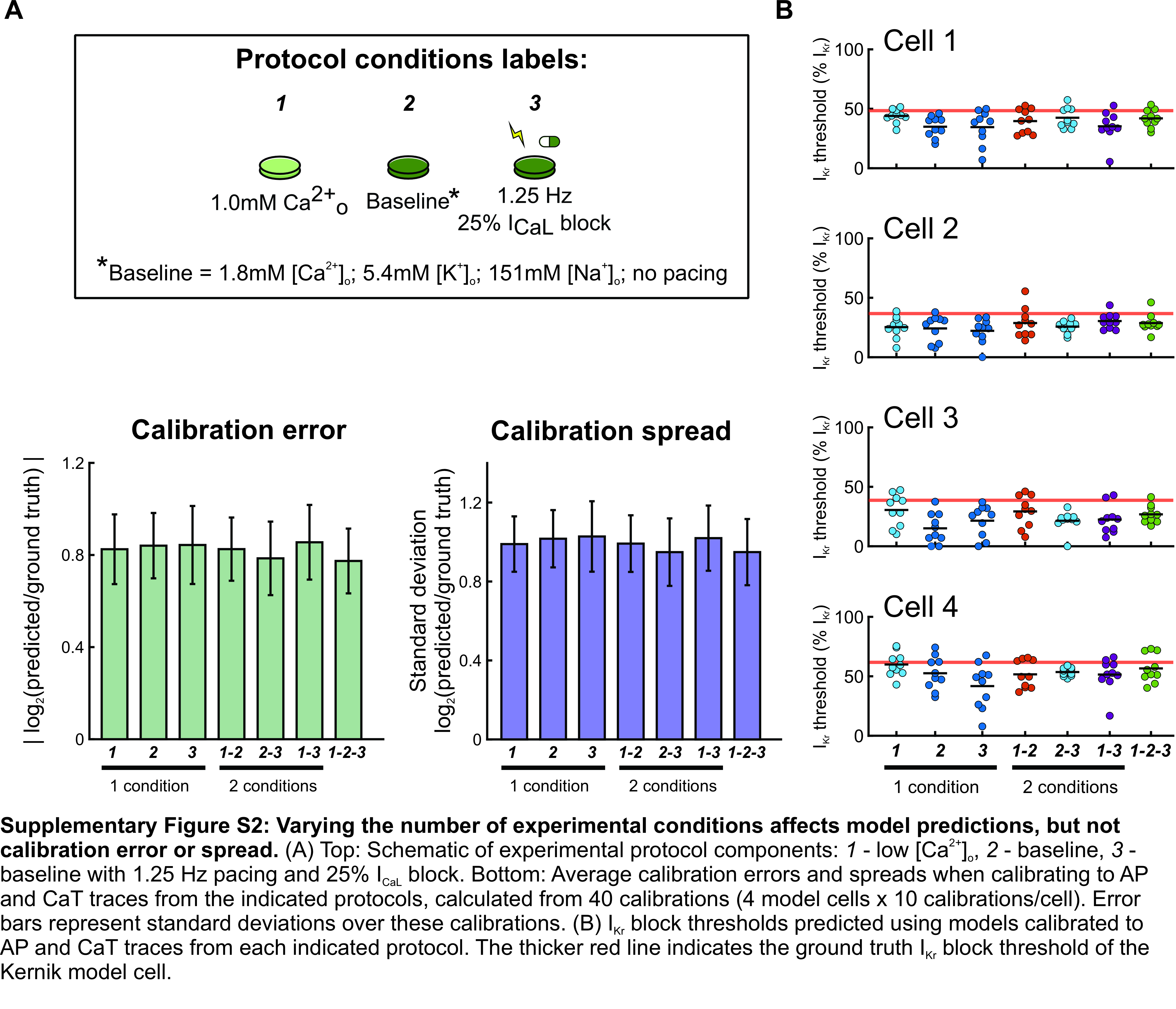

Supplement: S2 Fig — (A) Top: Schematic of experimental protocol components: 1—low [Ca2+]o, 2—baseline, 3—baseline with 1.25 Hz pacing and 25% ICaL block. Bottom: Average calibration errors and spreads when calibrating to AP and CaT traces from the indicated protocols, calculated from 40 calibrations (4 model cells x 10 calibrations/cell). Error bars represent standard deviations over these calibrations. (B) IKr block thresholds predicted using models calibrated to AP and CaT traces from each indicated protocol. The thicker red line indicates the ground truth IKr block threshold of the Kernik model cell. (TIF) [file pcbi.1011806.s002.tif]

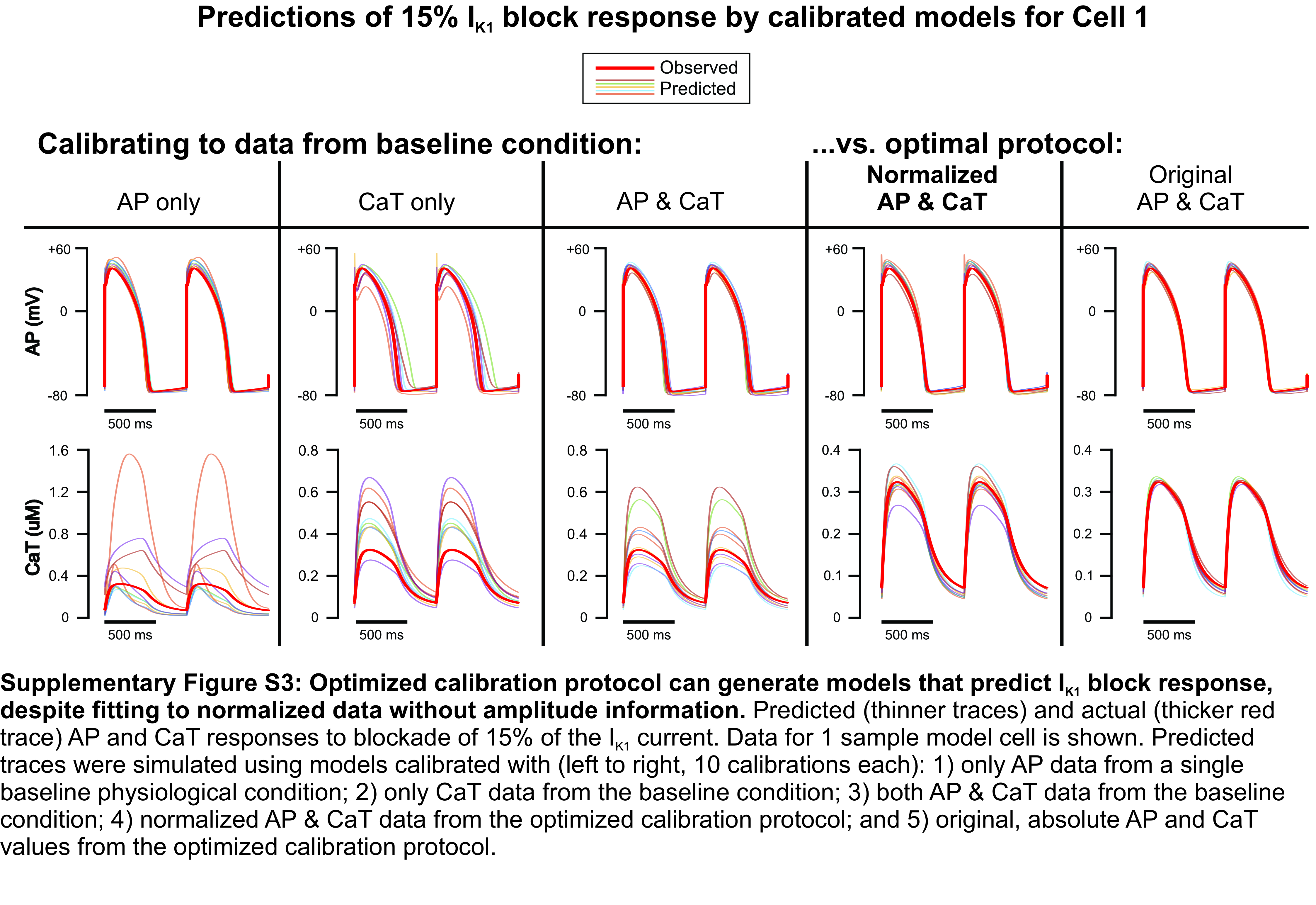

Supplement: S3 Fig — Predicted (thinner traces) and actual (thicker red trace) AP and CaT responses to blockade of 15% of the IK1 current. Data for 1 sample model cell is shown. Predicted traces were simulated using models calibrated with (left to right, 10 calibrations each): 1) only AP data from a single baseline physiological condition; 2) only CaT data from the baseline condition; 3) both AP & CaT data from the baseline condition; 4) normalized AP & CaT data from the optimized calibration protocol; and 5) original, absolute AP and CaT values from the optimized calibration protocol. (TIF) [file pcbi.1011806.s003.tif]

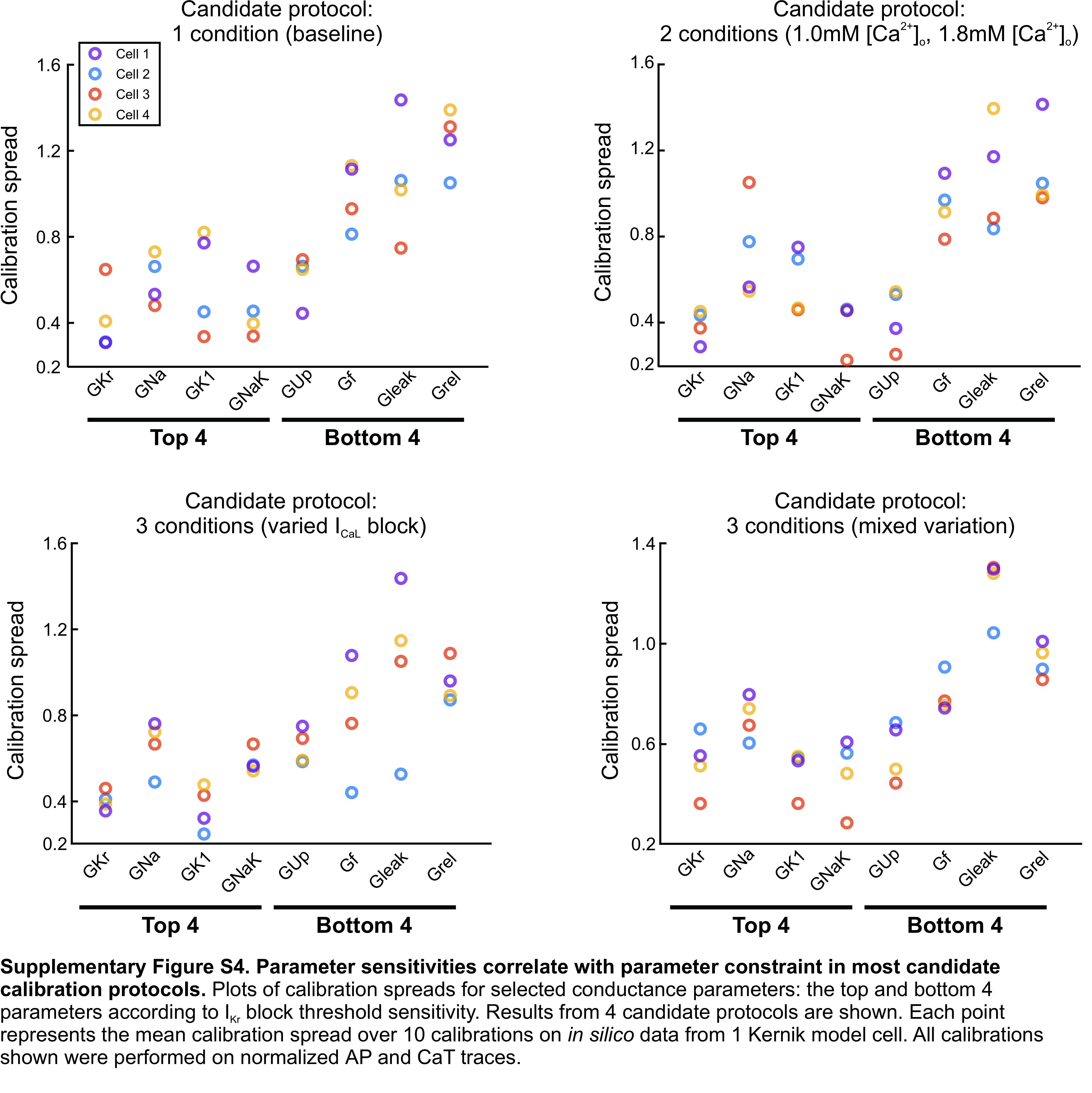

Supplement: S4 Fig — Plots of calibration spreads for selected conductance parameters: the top and bottom 4 parameters according to IKr block threshold sensitivity. Results from 4 candidate protocols are shown. Each point represents the mean calibration spread over 10 calibrations on in silico data from 1 Kernik model cell. All calibrations shown were performed on normalized AP and CaT traces. (TIF) [file pcbi.1011806.s004.tif]

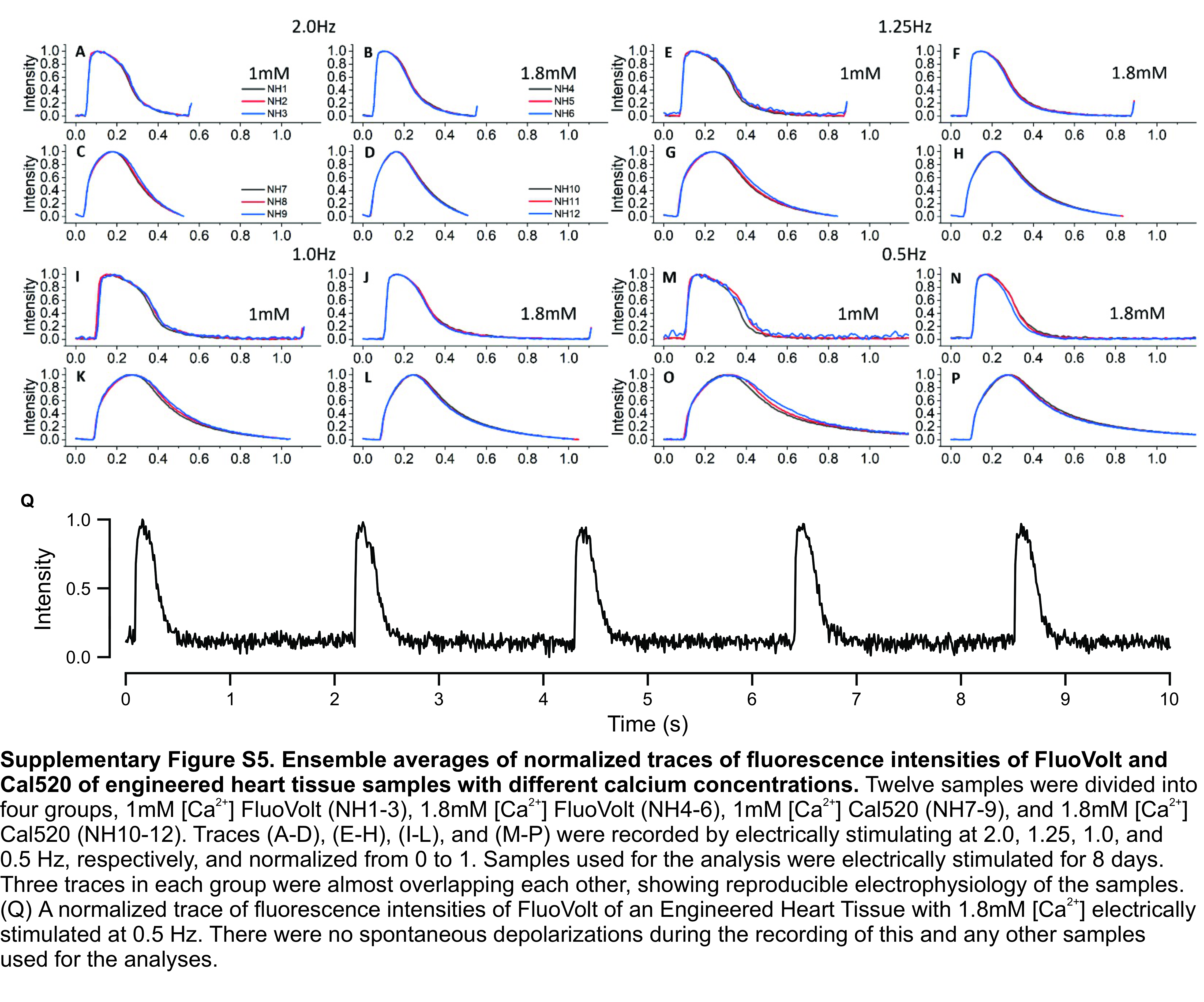

Supplement: S5 Fig — Twelve samples were divided into four groups, 1mM [Ca2+]o FluoVolt (NH1-3), 1.8mM [Ca2+]o FluoVolt (NH4-6), 1mM [Ca2+]o Cal520 (NH7-9), and 1.8mM [Ca2+]o Cal520 (NH10-12). Traces (A-D), (E-H), (I-L), and (M-P) were recorded by electrically stimulating at 2.0, 1.25, 1.0, and 0.5 Hz, respectively, and normalized from 0 to 1. Samples used for the analysis were electrically stimulated for 8 days. Three traces in each group were almost overlapping each other, showing reproducible electrophysiology of the samples. (Q) A normalized trace of fluorescence intensities of FluoVolt of an Engineered Heart Tissue with 1.8mM [Ca2+]o electrically stimulated at 0.5 Hz. There were no spontaneous depolarizations during the recording of this and any other samples used for the analyses. (TIF) [file pcbi.1011806.s005.tif]
