# Supplementary material for: Creating cell-specific computational models of stem cell-derived cardiomyocytes using optical experiments
Source: PLoS Comput Biol. 2024 Sep 11;20(9):e1011806. doi: 10.1371/journal.pcbi.1011806 (PMC11460686; doi:10.1371/journal.pcbi.1011806)
Supplement: S1 Table — Initial conditions were taken from the steady state values of a 10 minute simulation of the baseline model in 151 mM [Na+], 1.8 mM [Ca2+], 5.4 mM [K+], without stimulus. (DOCX) [file pcbi.1011806.s006.docx]

**S1 Table: Initial membrane potential, ion concentrations, channel states of the Kernik model.** Initial conditions were taken from the steady state values of a 10 minute simulation of the baseline model in 151 mM [Na^+^], 1.8 mM [Ca^2+^], 5.4 mM [K^+^], without stimulus.

| **Parameter** | **Units (if applicable)** | **Value** |
| --- | --- | --- |
| Membrane potential | millivolt (mV) | -75.7445 |
| Sarcoplasmic reticulum [Ca^2+^] | millimolar (mM) | 0.3390 |
| Cytosolic [Ca^2+^] | millimolar (mM) | 0.0002031 |
| Cytosolic [Na^+^] | millimolar (mM) | 7.1693 |
| Cytosolic ]K^+^] | millimolar (mM) | 104.7488 |
| Ca_ligand | millimolar (mM) | 0 |
| Activation in I_CaL_ (d) |  | 0.0003867 |
| Inactivation in I_CaL_ (f1) |  | 0.1659 |
| Ca^2+^-dependent inactivation in I_CaL_ (fCa) |  | 0.9271 |
| Activation in I_Kr_ (Xr1) |  | 0.3212 |
| Inactivation in I_Kr_ (Xr2) |  | 0.4522 |
| Activation in I_Ks_ (Xs) |  | 0.1578 |
| Inactivation in I_Na_ (h) |  | 0.7433 |
| Slow inactivation in I_Na_ (j) |  | 0.1211 |
| Activation in I_Na_ (m) |  | 0.02922 |
| Inactivation in I_f_ (Xf) |  | 0.006205 |
| Inactivation in I_to_ (s) |  | 0.7361 |
| Activation in I_to_ (r) |  | 0.0002641 |
| Activation in I_CaT_ (dCaT) |  | 0.0002636 |
| Inactivation in I_CaT_ (fCaT) |  | 0.7468 |
| RyR closed |  | 0.01223 |
| RyR open |  | 0.0001544 |
| RyR inactivated |  | 0.01232 |
| Stimulus | pA/pF | 0 |
