# Supplementary material for: Creating cell-specific computational models of stem cell-derived cardiomyocytes using optical experiments
Source: PLoS Comput Biol. 2024 Sep 11;20(9):e1011806. doi: 10.1371/journal.pcbi.1011806 (PMC11460686; doi:10.1371/journal.pcbi.1011806)
Supplement: S2 Table — Conductance parameter names and values of baseline Kernik model (first 2 columns), and the corresponding multiplier factors used to create the Kernik model cells in the final in silico dataset. (DOCX) [file pcbi.1011806.s007.docx]

**S2 Table: Maximal conductance parameters used to simulate the *in silico* dataset.** Conductance parameter names and values of baseline Kernik model (first 2 columns), and the corresponding multiplier factors used to create the Kernik model cells in the final *in silico* dataset.

|  | **Baseline** | **Cell 1** | **Cell 2** | **Cell 3** | **Cell 4** |
| --- | --- | --- | --- | --- | --- |
| **G_Na_** | 9.7206 nS/pF | 1.0013 | 0.8468 | 0.9188 | 1.0280 |
| **G_f_** | 0.0435 nS/pF | 1.1472 | 0.8221 | 1.0750 | 0.6829 |
| **G_CaL_** | 0.3080 nS/pF | 0.8428 | 0.7935 | 0.9320 | 0.9296 |
| **G_to_** | 0.1178 nS/pF | 0.8065 | 0.8988 | 1.0554 | 0.8440 |
| **G_Ks_** | 0.0077 nS/pF | 0.9820 | 0.6700 | 0.5988 | 0.8582 |
| **G_Kr_** | 0.2180 nS/pF | 0.9507 | 1.2127 | 1.0977 | 0.7981 |
| **G_K1_** | 0.1338 nS/pF | 1.2699 | 1.1096 | 1.4488 | 1.0158 |
| **G_PMCA_** | 0.2625 pA/pF | 1.1289 | 0.9960 | 1.2310 | 1.5240 |
| **G_bNa_** | 4.3500x10^-4^ nS/pF | 1.1142 | 0.9931 | 1.1998 | 0.8666 |
| **G_bCa_** | 3.6704x10^-4^ nS/pF | 0.7490 | 0.8525 | 0.9532 | 0.9454 |
| **G_up_** | 1.1050x10^-4^ mM/s | 0.8240 | 1.2260 | 1.0369 | 1.2628 |
| **G_rel_** | 12.500 ms^-1^ | 1.0412 | 0.9737 | 1.0501 | 1.2745 |
| **G_NCX_** | 1100.0 pA/pF | 0.9328 | 0.8668 | 1.0195 | 1.1020 |
| **G_NaK_** | 2.4761 pA/pF | 1.2944 | 1.3103 | 0.8470 | 1.2278 |
| **G_SRleak_** | 1.6000x10^-6^ ms^-1^ | 1.3076 | 0.9560 | 0.9320 | 1.1902 |
| **G_CaT_** | 0.1850 nS/pF | 0.8903 | 0.8889 | 0.9656 | 0.9265 |
