# Supplementary material for: Creating cell-specific computational models of stem cell-derived cardiomyocytes using optical experiments
Source: PLoS Comput Biol. 2024 Sep 11;20(9):e1011806. doi: 10.1371/journal.pcbi.1011806 (PMC11460686; doi:10.1371/journal.pcbi.1011806)
Supplement: S3 Table — A list of the various combinations of extracellular ion concentrations, pacing cycle lengths, and/or channel blocks simulated to generate the in silico dataset. (DOCX) [file pcbi.1011806.s008.docx]

**S3 Table: Experimental conditions included in simulated dataset.** A list of the various combinations of extracellular ion concentrations, pacing cycle lengths, and/or channel blocks simulated to generate the *in silico* dataset.

| **Condition** | **Stimulus?** | **Cycle length** | **[Na^+^]_extracellular_** | **[K^+^]_extracellular_** | **[Ca^2+^]_extracellular_** | **I_CaL_ block** | **I_Kr_ block** |
| --- | --- | --- | --- | --- | --- | --- | --- |
| 1 | Yes | 800 ms | 151 mM | 5.4 mM | 2.6 mM | 0% | 0% |
| 2 | No | N/A | 151 mM | 5.4 mM | 2.6 mM | 0% | 0% |
| 3 | Yes | 800 ms | 151 mM | 5.4 mM | 1.0 mM | 0% | 0% |
| 4 | No | N/A | 151 mM | 5.4 mM | 1.0 mM | 0% | 0% |
| 5 | Yes | 800 ms | 151 mM | 5.4 mM | 1.8 mM | 25% | 0% |
| 6 | No | N/A | 151 mM | 5.4 mM | 1.8 mM | 25% | 0% |
| 7 | Yes | 800 ms | 151 mM | 5.4 mM | 1.8 mM | 50% | 0% |
| 8 | No | N/A | 151 mM | 5.4 mM | 1.8 mM | 50% | 0% |
| 9 | Yes | 800 ms | 151 mM | 5.4 mM | 1.8 mM | 0% | 15% |
| 10 | No | N/A | 151 mM | 5.4 mM | 1.8 mM | 0% | 15% |
| 11 | Yes | 800 ms | 151 mM | 5.4 mM | 1.8 mM | 0% | 30% |
| 12 | No | N/A | 151 mM | 5.4 mM | 1.8 mM | 0% | 30% |
| 13 | Yes | 800 ms | 151 mM | 5.8 mM | 1.8 mM | 0% | 0% |
| 14 | No | N/A | 151 mM | 5.8 mM | 1.8 mM | 0% | 0% |
| 15 | Yes | 800 ms | 151 mM | 5.0 mM | 1.8 mM | 0% | 0% |
| 16 | No | N/A | 151 mM | 5.0 mM | 1.8 mM | 0% | 0% |
| 17 | Yes | 600 ms | 151 mM | 5.4 mM | 1.8 mM | 0% | 0% |
| 18 | Yes | 800 ms | 151 mM | 5.4 mM | 1.8 mM | 0% | 0% |
| 19 | No | N/A | 151 mM | 5.4 mM | 1.8 mM | 0% | 0% |
