# Supplementary material for: Creating cell-specific computational models of stem cell-derived cardiomyocytes using optical experiments
Source: PLoS Comput Biol. 2024 Sep 11;20(9):e1011806. doi: 10.1371/journal.pcbi.1011806 (PMC11460686; doi:10.1371/journal.pcbi.1011806)
Supplement: S4 Table — The GA search settings used for all model calibrations in this study. (DOCX) [file pcbi.1011806.s009.docx]

**S4 Table: Genetic algorithm settings.** The GA search settings used for all model calibrations in this study.

| **Setting** | **Value** | **Description** |
| --- | --- | --- |
| Initial population lower bound | -2 | Log_2_ scaled parameter multiplier |
| Initial population upper bound | 2 | Log_2_ scaled parameter multiplier |
| Initial population distribution | Uniform | Log_2_ scaled |
| Population size | 150 Kernik models |  |
| Number of generations | 20 |  |
| Selection function | Selection tournament, size 4 | Selects parent Kernik model as the highest fitness model out of 4 randomly chosen from the population |
| Crossover function | Scattered | Creates “child” Kernik model using a random binary vector of the same length as the number of parameters to be calibrated, selecting the parameter multiplier where the vector is a 1 from the first parent and the parameter multiplier where the vector is a 0 from the second parent. |
| Crossover fraction | 0.85 | Fraction of the next generation, other than elite children, that are produced by crossover |
| Mutation function | Adaptive feasible | Randomly generates mutation directions that are adaptive with respect to the last successful or unsuccessful generation. The mutation chooses a direction and step length that satisfies bounds and linear constraints |
| Elite count | 5% of population | Rounded up if non-integer |
| Fitness function evaluation | Parallel | Fitness function is evaluated in parallel environment |
