# Supplementary material for: Creating cell-specific computational models of stem cell-derived cardiomyocytes using optical experiments
Source: PLoS Comput Biol. 2024 Sep 11;20(9):e1011806. doi: 10.1371/journal.pcbi.1011806 (PMC11460686; doi:10.1371/journal.pcbi.1011806)
Supplement: S1 Text — Expanded abbreviations and definitions of several phrases commonly used throughout this study. (DOCX) [file pcbi.1011806.s010.docx]

**S1 Text: Glossary of abbreviations and terms used in this study.** Expanded abbreviations and definitions of several phrases commonly used throughout this study.

- **AP:** Action potential
- **CaT:** Calcium transient
- **GA:** Genetic algorithm
- **iPSC-CM:** Induced pluripotent stem cell-derived cardiomyocyte
- **G_x_, I_x_, J_x_:** Maximal conductance (G), current density (I), or flux (J) for **x**, where **x** can be: Na (Na^+^), f (funny Na^+^), CaL (L-type Ca^2+^), to (transient outward K^+^), Ks (slow delayed rectifier K^+^), Kr (rapid delayed rectifier K^+^), K1 (inward rectifier K^+^), PMCA (plasma membrane Ca^2+^ ATPase), bNa (background Na^+^), bCa (background Ca^2+^), Up (sarcoplasmic reticulum Ca^2+^ uptake), rel (sarcoplasmic reticulum Ca^2+^ release), NCX (Na^+^/Ca^2+^ exchanger), NaK (Na^+^/K^+^ ATPase), SRleak (sarcoplasmic reticulum leak Ca^2+^), or CaT (T-type Ca^2+^)
- **Model/parameter calibration**: tuning one or more parameters in a computational model such that the model output more closely matches experimental data
- **Experiment/protocol optimization**: the process of determining what type and amount of data is sufficient but also feasible for our model calibration goals
  - Protocol conditions – buffer calcium, potassium, or sodium concentrations; addition or removal of stimulus; pacing rates; channel block; etc.
  - Protocol length – number of protocol conditions
  - Protocol data type – AP, CaT, or both; normalized or non-normalized data
- **Model prediction**: using the calibrated computational model to simulate responses to novel conditions, drugs, or perturbations that were not used for calibration
- **Computational pipeline**: the full process of iPSC-CM computational model calibration; includes iPSC-CM data acquisition/simulation, data processing, parameter calibration using genetic algorithm, validation of calibrated models on an unseen condition (i.e. evaluating model predictions)
